# Increased LV apical untwist during preload reduction in healthy humans: an echocardiographic speckle tracking study during lower body negative pressure

**DOI:** 10.14814/phy2.12330

**Published:** 2015-03-22

**Authors:** Anders Hodt, Jonny Hisdal, Marie Stugaard, Einar Stranden, Dan Atar, Kjetil Steine

**Affiliations:** 1 Department of Cardiology B, Institute of Clinical Medicine, Oslo University HospitalOslo, Norway; 2 Section of Vascular Investigations, Oslo University HospitalOslo, Norway; 3 Faculty of Medicine, University of OsloOslo, Norway; 4 Department of Cardiology, Hyogo Cancer CenterAkashi, Japan; 5 Department of Cardiology, Akershus University HospitalOslo, Norway

**Keywords:** Diastole, echocardiography, myocardial deformation, preload reduction, speckle tracking imaging

## Abstract

We sought to investigate the effect of reduced preload on left ventricle (LV) untwist and early diastolic filling in healthy individuals. Twelve healthy men, 22 (22, 23) years of age, were examined at rest and during applied lower body negative pressure (LBNP) of −20 mmHg and −40 mmHg, respectively. Regional untwist and untwist rate during IVRT were calculated at LV basal, papillary, subpapillary, and apical short axis levels by two dimensional speckle tracking echocardiography. Left ventricle early diastolic filling was assessed by transmitral E-wave (E) peak velocity by pulsed Doppler and flow propagation velocity (*V*p) by color M-mode Doppler and early diastolic pulsed Doppler tissue velocities (E') from septal and lateral mitral annulus. From rest to LBNP −40 mmHg, the LV untwist and untwist rate at subpapillary level increased from 2.3 (1.4, 3.5) to 4.5 (3.1, 7.6) degrees and from −36 (−51, −25) to −69 (−127, −42) °/s (*P* < 0.001, *P* = 0.003), respectively, while apical untwist and untwist rate increased from 3.9 (2.3, 4.3) to 7.6 (6.4, 10.5) degrees and from −51 (−69, −40) to −118 (−170, −84) °/s (*P* < 0.001, *P* < 0.001), respectively. Since untwist and untwist rate at the basal level were unchanged, this created markedly larger base to apical untwist and untwist rate gradients from rest to LBNP −40 mmHg. E, *V*p, and E' were reduced by 34, 32, and 39%, respectively. LV untwist and untwist rate during IVRT were increased at apical levels, which might be a physiological mechanism to minimize the impairment in LV early diastolic filling during preload reduction.

## Introduction

As a result of nonuniform oppositely and obliquely directed fiber orientation (Streeter et al. 1969) the left ventricle (LV) performs a global wringing motion during ejection, called net twist, by apical counterclockwise and basal clockwise rotation (Arts et al. 1984; Buchalter et al. 1990; Lorenz et al. 2000). In healthy humans, magnetic resonance imaging (MRI) has demonstrated, by recording five to six short axis levels, regional LV gradients with increasing degree of counterclockwise rotation towards apex during systole (Lorenz et al. 2000). During diastole the LV performs a global net untwist that mainly occurs during isovolumic relaxation time (IVRT) (Rademakers et al. 1992). More recently, it has been shown that LV untwist during IVRT plays an important role for events during the time interval of early diastolic filling (Wang et al. 2007; Notomi et al. 2008; Burns et al. 2010; Opdahl et al. 2012).

The effect of reduced preload on net twist has shown conflicting results in animal and human studies (Kroeker et al. 1995; Dong et al. 1999; Burns et al. 2010; Park et al. 2010; Opdahl et al. 2012). Our group has shown in healthy humans that reduced preload by lower body negative pressure (LBNP) technique elicited increased global net twist and regional counterclockwise rotation in the apical levels at end-systole by recording four short axis levels using two dimensional speckle tracking echocardiography (STE) (Hodt et al. 2011). We suggested that increased net twist and apical rotation could be physiological compensatory mechanisms of the LV to minimize the reduction in stroke volume, and thus maintain arterial blood pressure, during reduction in preload.

Left ventricle net untwist during IVRT by preload reduction has been studied in animal (Kroeker et al. 1995; Wang et al. 2007; Opdahl et al. 2012) and human (Park et al. 2010) studies with inconsistent results. To the best of our knowledge, however, studies showing the impact of preload reduction on changes in LV regional untwist in humans have so far not been performed.

The aim of this study was therefore by recording several LV short-axis levels using STE to investigate the impact of reduced preload by LBNP on regional and global changes in untwist and untwist rate during IVRT in healthy individuals. Our hypothesis was that untwist would increase, reflecting myocardial compensation to reduced preload. Additionally, we wanted to study the impact of regional LV untwist on events during early diastolic filling.

## Materials and Methods

### Study subjects

In a previous study, systolic function during reduced preload was investigated in 12 healthy male students (Hodt et al. 2011). In this study, the STE data were reanalyzed, but now focusing on diastolic function in the same 12 students (age 22 (2.3) years, height 183 (0.1) cm, weight 80 (9.5) kg, and body mass index 24 (1.9) kg/m (median (IQR)). All subjects were nonsmokers without any history of cardiovascular disease or any medication. Written informed consent was obtained from each subject at inclusion. The study was approved by the regional committee for medical research ethics.

### Lower body negative pressure (LBNP)

The LBNP technique was applied by a custom-built chamber and pressure-control system previously described in detail (Hisdal et al. 2001; Hodt et al. 2011).

### Study design

**Figure 1:**
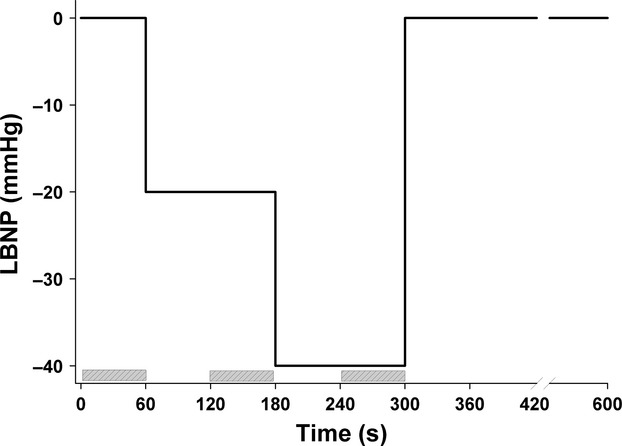
Schematic overview of the experimental setup showing different levels (*y*-axis) and duration (*x*-axis) of preload reduction by lower body negative pressure (LBNP) in one of the eight experiments performed in each of the 12 subjects. The time period of the echocardiographic recordings is drawn with marked boxes.

The study was carried out at Oslo University Hospital, Aker, Norway. To obtain hemodynamic and echocardiographic data, eight repetitive and identical experiments were performed during LBNP in each subject (Fig.1). The interval between the duration of the LBNP was 3–5 min and started when the hemodynamic variables were returned to baseline levels (Hisdal et al. 2001). The subjects were placed comfortably on a bench with the lower body inside the LBNP chamber and were acclimated in a resting position for 10 min. Then, each experiment started with a period of 1 min with atmospheric pressure in the chamber, defined as rest. Pressure in the chamber was then gradually lowered over 15 sec to −20 mmHg. This pressure level was kept for 2 min including one initial minute to reach steady-state level of stroke volume (SV), heart rate (HR), cardiac output (CO), and mean arterial pressure (MAP). After 2 min, the pressure was further gradually lowered over 15 sec to LBNP −40 mmHg, and this was held for another 2 min. During the last minute at LBNP −20 and −40 mmHg, the hemodynamic or echocardiographic recording was done. Finally, the pressure was turned back to atmospheric level over 5–10 sec. During each experiment, HR, MAP, and three-lead surface ECG were continuously recorded. MAP was acquired by measuring finger arterial pressure by a photoplethysmographic recording device (Finometer, FMS Finapres Measurement Systems, Arnhem, Netherlands).

### Hemodynamic and echocardiographic recordings

In the first experiment, beat-to-beat SV and CO were recorded from the suprasternal notch toward the aortic root, and the sample volume range was adjusted so that measurements were made 1–2 cm above the aortic valve using a bidirectional ultrasound pulsed Doppler velocimeter (SD-100, GE Vingmed Ultrasound, Horten, Norway) with a 2 MHz handheld transducer as described previously (Hisdal et al. 2001). In experiment numbers 2–8 echocardiographic data were sampled by a Vivid 7 ultrasound scanner with a 1.7–2.5 MHz transducer (GE Vingmed Ultrasound, Horten, Norway). To optimize and standardize image acquisition, all recordings were performed at end-expiration with subjects in supine left lateral position. In the second to the fifth experiment, three-two-dimensional (2D) loops (67–82 frames/second (FPS)) were recorded. To be able to show base-apex gradients, different LV short-axis levels (basal, papillary, subpapillary, and apical) were recorded for the analysis of regional rotation and changes of untwist during the IVRT period**.** Effort was made to handhold the probe stable at the same location on the chest during each experiment, from start of the rest period to end of LBNP −40 mmHg, in order to limit variations of recording of different LV short-axis levels. Each level was standardized to reflect four LV landmarks as follows: The basal landmark was acquired from a standard parasternal transducer position, where mitral valve leaflets were observed in the LV cavity. From a more anterolateral transducer position, the apical level was recorded just proximal to the level that showed luminal closure at end-systole. The papillary and subpapillary levels were recorded from anterior positions between the basal and apical projections. The papillary level was defined where both papillary muscles were seen at maximum sizes, and the subpapillary level was defined just inferior to the papillary muscles. All loops were recorded with the probe perpendicular to the LV longitudinal axis, achieving the LV cross-section as circular as possible.

In experiment numbers 6, 7, and 8, events during early diastolic filling were recorded. The transmitral early diastolic peak velocity (E) was obtained by pulsed Doppler, flow propagation velocity (*V*p) by color M-mode Doppler and early diastolic velocity (E') by tissue Doppler echocardiography (TDE) data (160 FPS).

### Analyses of LV untwist, untwist rate, and early diastolic filling

By the use of 2D STE, rotation and rotation rate can be derived and analyzed offline (EchoPAC, version 8.1, GE Vingmed Ultrasound, Horten, Norway) by following specific acoustic speckles in the 2D myocardium frame by frame throughout the cardiac cycle (Bohs and Trahey 1991). Rotation and rotation rate at each short-axis loop was analyzed by manually tracing LV endocardium on a frame with well-defined border during late systole. A region of interest of the LV was adjusted to include most of myocardium but not pericardium. Tracking quality was scored automatically by the software as acceptable or not acceptable. The observer readjusted the endocardial trace line until all six predefined LV segments (septal, antero-septal, anterior, lateral, posterior, and inferior) were scored as acceptable. If one or several segments were still scored as nonacceptable, these segments were excluded. Rotation and rotation rate at each frame throughout the heart cycle were automatically calculated by the software algorithm in six different segments. The start point of the integration was adjusted to the ECG signal corresponding to time of end-diastole. Finally, an average curve from all six segments was automatically calculated as an estimate of rotation and rotation rate at that particular short-axis level. The average curve from the second of the three recorded 2D loops was used in the further analysis. The reason for this was that the second of the three 2D loops always contained the time of end-diastole, defined by color anatomic M-mode (CAMM). It would have been possible to use mean of the second and the third loop. However, all measurements were made in the steady-state period where the variation between different heart cycles was minimal. We therefore decided only to present values from one heart cycle. The same procedure was repeated for each of the four short-axis levels. The regional rotation and rotation rate data at the four short-axis levels with corresponding time data were numerically decoded into a file and transferred to a spreadsheet program (Microsoft Office Excel).

Peak value of systolic rotation was measured at basal and apical levels. Since rotation is zero or close to zero degree at papillary level and sometimes without a definite peak value at subpapillary level, rotation at time of end-systole was measured at these levels (Hodt et al. 2011).

Regional untwist at basal and apical levels was calculated as peak systolic rotation (°) – rotation at time of mitral valve opening (MVO) (°). At papillary and subpapillary levels regional untwist was calculated as rotation at end-systole (°) – rotation at time of MVO (°). Untwist rate (°/s) at each short-axis level was measured at the first peak after end-systole and typically during the time-interval of IVRT. Left ventricle net twist and net untwist, reflecting the wringing motion of the left ventricle, during systole and diastole, respectively. Left ventricle net twist was calculated as peak systolic apical rotation (°) – peak systolic basal rotation (°). Net untwist was calculated as net twist at end-systole (°) – net untwist at time of MVO (°).

Early diastolic filling by *V*p was assessed by tracking a slope of the first aliasing velocity from the mitral valve plane in early diastole to 4 cm into the left ventricle, cm/sec (Garcia et al. 2000). E' was calculated by averaging the E' at the septal and lateral part of the mitral annulus. Transmitral peak E, *V*p, and E' were measured in three consecutive beats and averaged.

The time of end-diastole, end-systole, and mitral valve opening of each subject were determined by the use of color anatomical M-mode data derived from TDE at the base of LV septum in apical four chamber view (Lind et al. 2004). By the use of color anatomical M-mode, these time points could be extracted by color shifts reflecting changes of myocardial velocities in the LV septum, which were related to QRS signals of the ECG. Time of end-diastole corresponded to start of the heart cycle (referenced as 0 ms).

### Statistical analysis

The results in the present manuscript are based on reanalysis of the echocardiographic recordings from the experiments presented on systolic function published in 2011 (Hodt et al. 2011). Due to reanalysis of also the systolic data, there may be minor differences between the results presented in the current paper and some of the figures presented in the previous study (Hodt et al. 2011). In the present analyses of diastolic function, not all variables showed normal distribution. The values are therefore presented as median and interquartile range (IQR). Friedman repeated measures analysis of variance on ranks, two sided, by the use of multiple comparisons (Tukey) was applied to test for changes between rest, LBNP −20 and LBNP −40 mmHg (SigmaPlot version 12, Systat Software, Inc, Germany). Reproducibility was tested in 12 subjects by two different observers, who independently measured peak untwist rate during IVRT at the LV apical level at rest, LBNP −20 and −40 mmHg. The variability was assessed by the ANOVA repeated measures test and expressed by the intraclass correlation coefficient (ICC) and 95% confidence interval (SPSS version 18, Chicago, IL, USA). A value of *P* < 0.05 was considered statistically significant.

## Results

Among the 23 screened subjects, 11 were excluded, due to noise or reverberations in two or more LV short-axis levels (Burns et al. 2010), <15% reduction of SV from rest to LBNP −40 mmHg (Berk et al. 1990) and symptoms of presyncope (Arbab-Zadeh et al. 2004). Finally, 12 subjects, age 22 (21, 23) years, height 183 (181, 187) cm, weight 80 (77, 84) kg, and body mass index 24 (23, 24) were included in the study. 2D loops by echocardiography at different short-axis levels were of good quality in all 12 subjects. However, due to paradoxical 2D movement of the antero-septal wall and noise from lung-tissue during LBNP, loops at basal and papillary level in one subject and at subpapillary level in another subject were excluded. Therefore, data at basal, papillary, and subpapillary levels represent 11 subjects and at apical level 12 subjects. The data from the papillary level are not presented, since the rotation is close to zero, and thus do not have any impact on our main results (Hodt et al. 2011).

### Hemodynamic data during LBNP

Hemodynamic and LV geometric data are listed in Table1. Time of end-systole was 360 (350, 380), 350 (340, 370) and 330 (320, 350) ms and time of mitral valve opening was 460 (440, 480), 460 (430, 470) and 440 (410, 450) ms at rest, LBNP −20 and −40 mmHg, respectively.

**Table 1:** Hemodynamic and geometric data at rest and during reduced preload by lower body negative pressure (LBNP) at −20 mmHg and −40 mmHg

(*n* = 12)	Rest	LBNP −20 mmHg	LBNP −40 mmHg	*P*-value
LV EDV, mL	154 (131, 175)	125 (118, 162)*	105 (104, 142)*^,^†	<0.001
LV ESV, mL	61 (54, 72)	56 (49, 64)	53 (47, 57)*^,^†	<0.001
LV global EF, %	61 (55, 63)	59 (54, 62)	58 (50, 61)*	=0.009
SV, mL/beat	82 (77, 98)	72 (64, 86)*	59 (49, 65)*^,^†	<0.001
HR, beat/min	52 (46, 58)	54 (45, 60)	61 (50, 69)*^,^†	<0.001
CO, mL/min	4.6 (3.7, 5.0)	3.9 (3.4, 4.4)	3.6 (2.9, 4.0)*	<0.001
MAP, mmHg	84 (79, 92)	84 (78, 91)	85 (82, 90)	0.34
TPR, mmHg, min/L	18 (17, 22)	21 (20, 26)	24 (22, 29)*	<0.001
LV ED length, cm	9.0 (8.9, 9.0)	8.6 (8.2, 9.0)*	8.4 (8.2, 8.9)*	<0.001
LV ED base diam, cm	5.5 (5.1, 5.8)	5.2 (4.8, 5.4)	4.7 (4.6, 5.2)*	<0.001

LV, left ventricle; EDV, end-diastolic volume; ESV, end-systolic volume; EF, ejection fraction; SV, stroke volume; HR, heart rate; CO, cardiac output; MAP, mean arterial pressure; TPR, total peripheral resistance; ED, end-diastole; diam, diameter.

Values are Median (IQR).

*P < 0.05 versus rest, ^†^*P* < 0.05 versus LBNP −20 mmHg.

### LV regional untwist and untwist rate during LBNP

**Figure 2:**
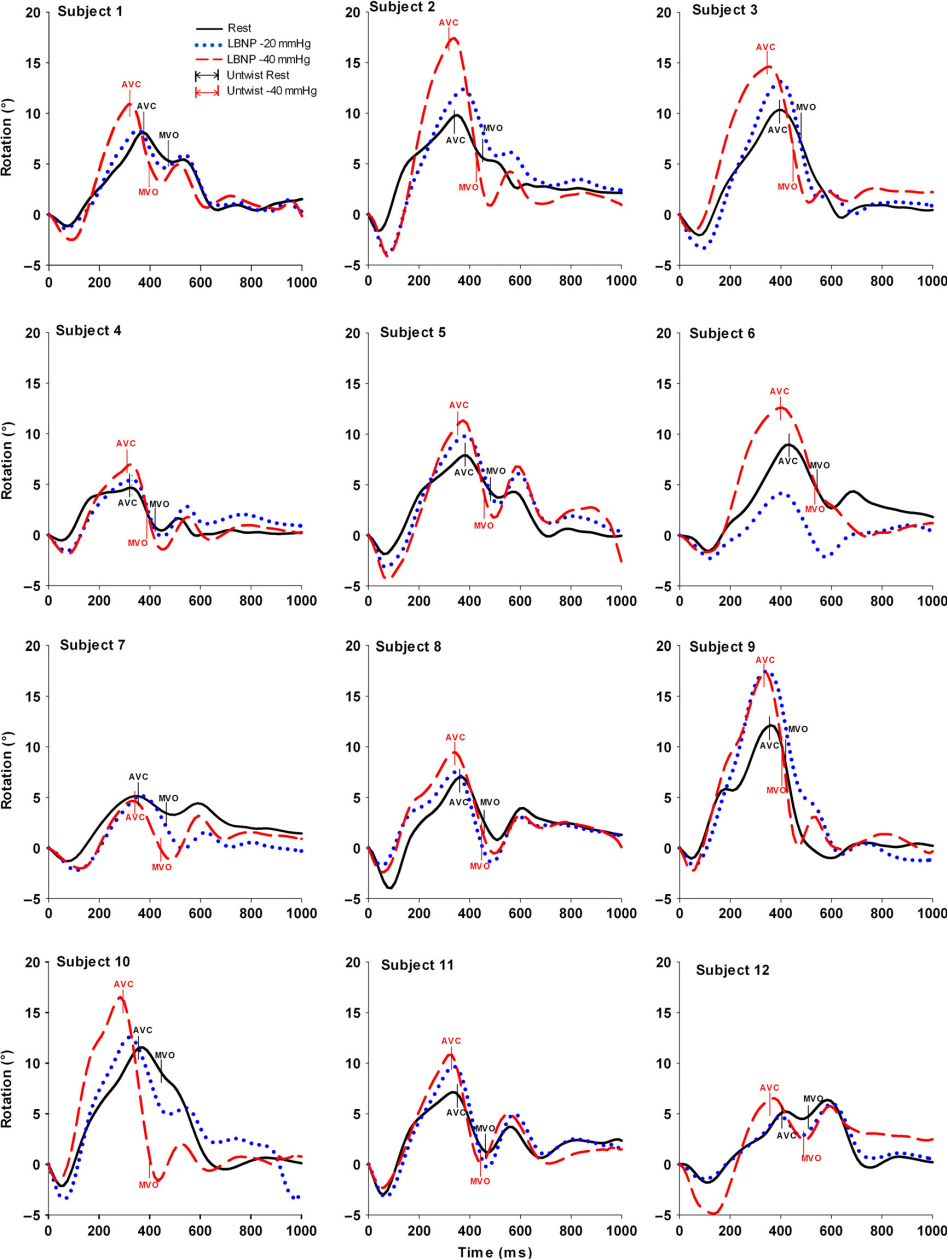
Apical rotation in all 12 healthy subjects at rest (solid line), at lower body negative pressure (LBNP) −20 mmHg (dotted line) and −40 mmHg (middle dash line). Upwards and downwards directed curves (values) correspond to counterclockwise and clockwise rotation, respectively. Time 0 represents end-diastole and time points of aorta valve closure (AVC) and mitral valve opening (MVO) are presented at rest and LBNP −40 mmHg.

**Figure 3:**
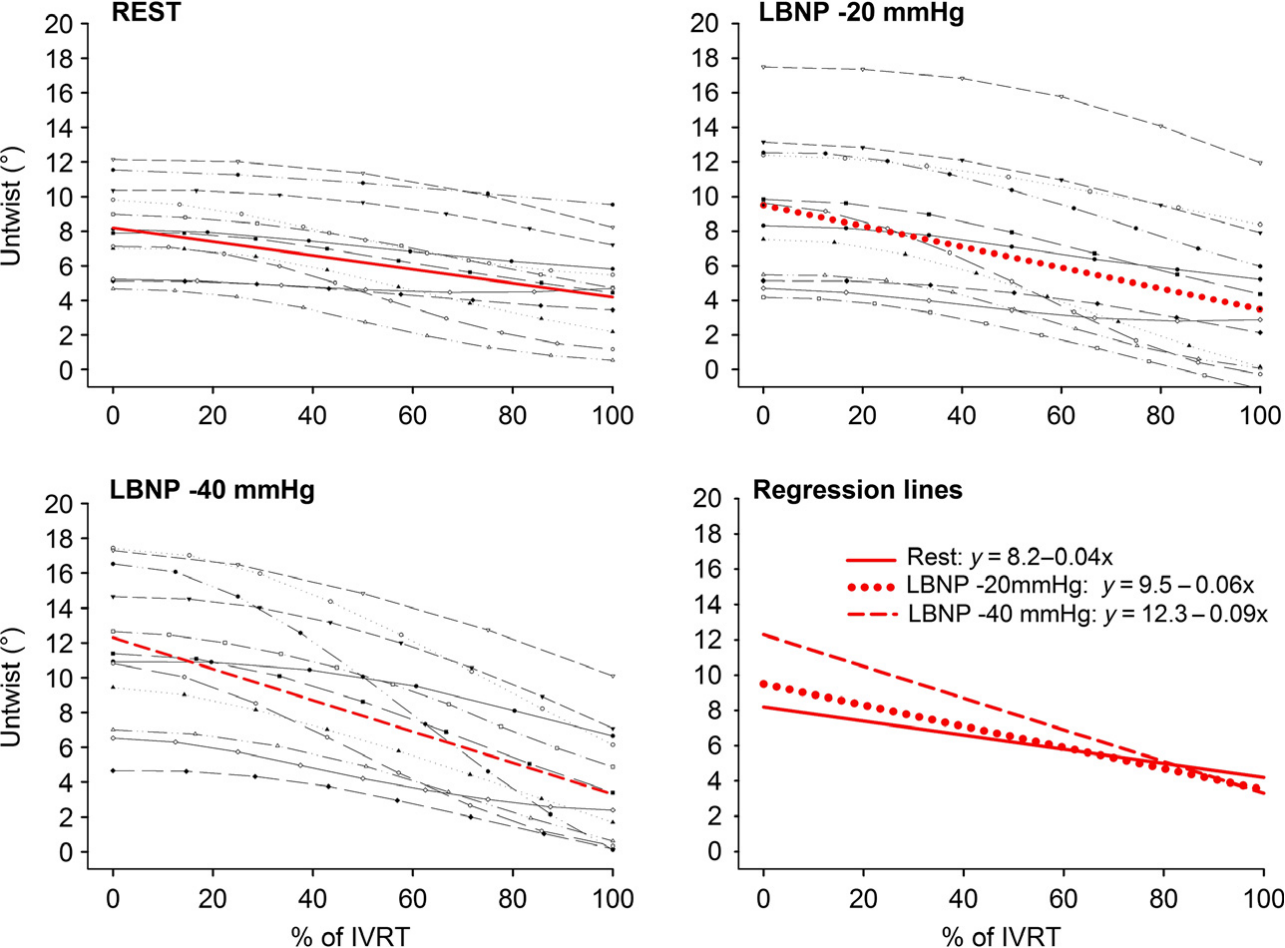
Apical untwist are plotted per frame during isovolumic relaxation time (IVRT) from end-systole (0%) to mitral valve opening (100%) for all subjects at rest, lower body negative pressure (LBNP) −20 and LBNP −40 mmHg and a regression line is calculated.

Left ventricle untwist during IVRT at the subpapillary and apical levels increased significantly from rest to LBNP −40 mmHg (*P* < 0.001, *P* < 0.001), respectively, while it was unchanged at basal level (*P* = 0.53) (Table2). The individual data showed an increase of LV untwist during IVRT at apical level in 12 of 12 subjects from rest to LBNP −40 mmHg (Figs2 and 3).

**Table 2:** Regional systolic rotation and untwist data during isovolumic relaxation time (IVRT) at rest and lower body negative pressure (LBNP) at −20 mmHg and −40 mmHg. Untwist was defined as: (peak systolic rotation – rotation at mitral valve opening (MVO)

	Rest	LBNP −20 mmHg	LBNP −40 mmHg	*P*-value
Basal level (*n* = 11)				
Peak sys. rotation, °	−5.4 (−6.8, −3.7)	−5.0 (−6.2, −4.5)	−5.4 (−7.0, −3.7)	0.70
Rotation, MVO, °	−2.6 (−4.7, −1.1)	−2.9 (−4.2, −0.9)	−2.7 (−4.5, −1.7)	0.70
Untwist, °	−2.0 (−3.7, −1.0)	−2.0 (−4.1, −1.2)	−1.8 (−3.4, −1.3)	0.53
Subpapillary level (*n* = 11)				
Rotation, ES, °	2.9 (1.4, 5.6)	3.2 (1.8, 7.1)	4.5 (1.4, 9.4)	0.06
Rotation, MVO, °	−0.0 (−1.1, 4.1)	0.2 (−2.7, 4.5)	−1.1 (−2.1, 2.3)	0.23
Untwist, °	2.3 (1.4, 3.5)	3.0 (1.8, 4.5)	4.5 (3.1, 7.6)*†	<0.001
Apical level (*n* = 12)				
Peak sys. rotation, °	8.1 (7.0, 10.3)	9.6 (5.5, 12.5)	11.4 (9.4, 16.5)*	<0.001
Rotation, MVO, °	4.7 (2.2, 7.2)	4.4 (0.1, 7.9)	3.4 (0.3, 6.7)	0.53
Untwist, °	3.9 (2.3, 4.3)	5.4 (4.0, 6.6)	7.6 (6.4, 10.5)*^,^†	<0.001

ES, end-systole.

Values are Median (IQR).

*P < 0.05 versus rest, ^†^*P* < 0.05 versus LBNP −20 mmHg.

Left ventricle untwist rate increased significantly at subpapillary and apical levels (*P* = 0.003, *P* < 0.001) but was unchanged at the basal level (*P* = 0.34) (Table3). Note that the untwist rate at subpapillary and apical levels increased 91 and 131% from rest to LBNP −40 mmHg, respectively, while the corresponding increase in rotation rate at subpapillary and apical levels was 23 and 34%, respectively (Table3).

**Table 3:** Regional systolic rotation rate (RR) and untwist rate (UTR) during isovolumic relaxation time (IVRT) at rest and lower body negative pressure (LBNP) at −20 mmHg and −40 mmHg

	Rest	LBNP −20 mmHg	LBNP −40 mmHg	*P*-value
Basal level (*n* = 11)				
Peak systolic RR, °/sec	−60 (−71, −52)	−59 (−71, −50)	−65 (−74, −44)	0.91
Time to peak sys. RR, ms	223 (209, 247)	226 (223, 254)	231 (223, 255)	0.60
Peak UTR, °/sec	61 (43, 83)	53 (32, 79)	48 (43, 67)	0.34
Time to peak UTR, ms	458 (445, 494)	453 (419, 484)	429 (401, 445)*	0.042
Subpapillary level (*n* = 11)				
Peak systolic RR, °/sec	45 (31, 70)	48 (33, 79)	55 (33, 93)	0.76
Time to peak sys. RR, ms	131 (105, 148)	129 (105, 157)	120 (118, 179)	0.60
Peak UTR, °/sec	−36 (−51, −25)	−36 (−65, −27)	−69 (−127, −42)*^,^†	0.003
Time to peak UTR, ms	431 (386, 456)	419 (406, 471)	406 (380, 432)*^,^†	0.006
Apical level (*n* = 12)				
Peak systolic RR, °/sec	64 (51, 73)	73 (59, 96)	86 (63, 113)*	0.003
Time to peak sys. RR, ms	135 (120, 196)	150 (120, 196)	157 (135, 202)*	0.039
Peak UTR, °/sec	−51 (−69, −40)	−79 (−103, −55)*	−118 (−170, −84)*^,^†	<0.001
Time to peak UTR, ms	431 (419, 449)	434 (413, 463)	413 (377, 434)*^,^†	0.003

Values are Median (IQR).

*P < 0.05 versus rest, ^†^*P* < 0.05 versus LBNP −20 mmHg.

### LV net untwist and untwist rate during LBNP

**Figure 4:**
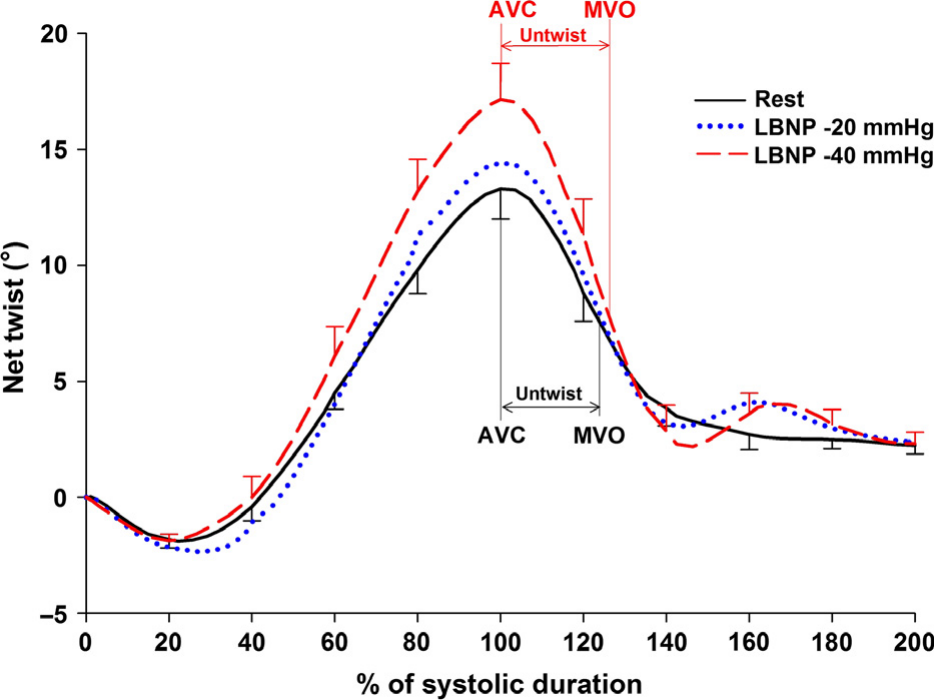
Average net LV twist and untwist in 11 subjects at rest, lower body negative pressure (LBNP) −20 and LBNP −40 mmHg. Normalized time is on the *x*-axis, when 0% represents end-diastole and 100% represents time of aorta valve closure (AVC). Time for AVC and mitral valve opening (MVO) are shown by vertical lines.

Left ventricle net untwist during IVRT increased significantly from rest to LBNP −20 and −40 mmHg, reflecting an increase in base to apex untwist gradient (Table4 and Fig.4). The individual data showed an increase of net LV untwist in 11 of 11 subjects from rest to LBNP −40 mmHg. Net LV peak untwist rate increased significantly from rest to LBNP −40 mmHg (Table5).

**Table 4:** Net twist and net untwist data at rest and lower body negative pressure (LBNP) at −20 mmHg and −40 mmHg. Net untwist was defined as: apical (rotation at ES – rotation at MVO) minus basal (rotation at ES – rotation at MVO)

(*n* = 11)	Rest	LBNP −20 mmHg	LBNP −40 mmHg	*P*-value
Net twist, ES, °	13.4 (11.9, 17.3)	15.2 (11.5, 18.1)	16.5 (13.4, 21.7)*^,^†	0.003
Net twist, MVO, °	8.0 (4.7, 11.2)	6.3 (1.4, 12.6)	6.6 (2.6, 11.4)	0.31
Net untwist, °	5.2 (4.7, 7.2)	6.7 (5.2, 9.7)*	9.2 (6.0, 11.4)*	<0.001

ES, end-systole; MVO, mitral valve opening.

Values are Median (IQR).

*P < 0.05 versus rest, ^†^*P* < 0.05 versus LBNP −20 mmHg.

**Table 5:** Net systolic twist rate (TR) and net untwist rate (UTR) during isovolumic relaxation time (IVRT) at rest and at lower body negative pressure (LBNP) of −20 mmHg and −40 mmHg. Net untwist rate during IVRT was calculated as the difference of untwist rate at apical level minus untwist rate at the basal level

(*n* = 11)	Rest	LBNP −20 mmHg	LBNP −40 mmHg	*P*-value
Net peak TR, °/sec	79 (68, 92)	89 (68, 126)*	124 (90, 140)*	0.006
Time peak TR, ms	236 (209, 275)	224 (209, 249)	219 (207, 236)*	0.042
Net peak UTR, °/sec	−103 (−130, −66)	−123 (−142, −103)	−148 (−180, −142)*	0.006
Time peak UTR, ms	445 (419, 458)	438 (406, 484)	419 (393, 438)*	0.020

Values are Median (IQR).

*P* < 0.05 versus rest.

### Early diastolic filling and apical untwist during LBNP

Early diastolic filling data at rest and at LBNP −20 and −40 mmHg are listed in Table6.

**Table 6:** Transmitral peak early E-wave (E) diastolic inflow velocity, mitral to apical flow propagation (*V*p), peak early (E') diastolic myocardial velocity at base and E/E' ratio at rest and lower body negative pressure (LBNP) at −20 mmHg and −40 mmHg

(*n* = 12)	Rest	LBNP −20 mmHg	LBNP −40 mmHg	*P*-value
E, cm/sec	70 (63, 78)	51 (50, 65)*	46 (41, 52)*	<0.001
Time E, ms	530 (515, 550)	530 (520, 550)	530 (515, 545)	0.53
*V*p, cm/sec	53 (45, 58)	42 (36, 46)*	36 (31, 42)*^,^†	<0.001
E', cm/sec	11.6 (10.0, 12.5)	10.0 (7.1, 11.0)*	7.1 (6.2, 8.7)*^,^†	<0.001
Time E', ms	520 (500, 550)	530 (500, 550)	500 (480, 530)*^,^†	0.005
E/E'	5.7 (5.1, 7.2)	5.8 (4.9, 7.1)	6.8 (5.4, 8.2)	0.44

E, transmitral early E-wave velocity, *V*p, flow propagation velocity, E', LV early diastolic velocity of the mitral annulus (septal + lateral/2).

Values are Median (IQR).

*P < 0.05 versus rest, ^†^*P* < 0.05 versus LBNP −20 mmHg.

**Figure 5:**
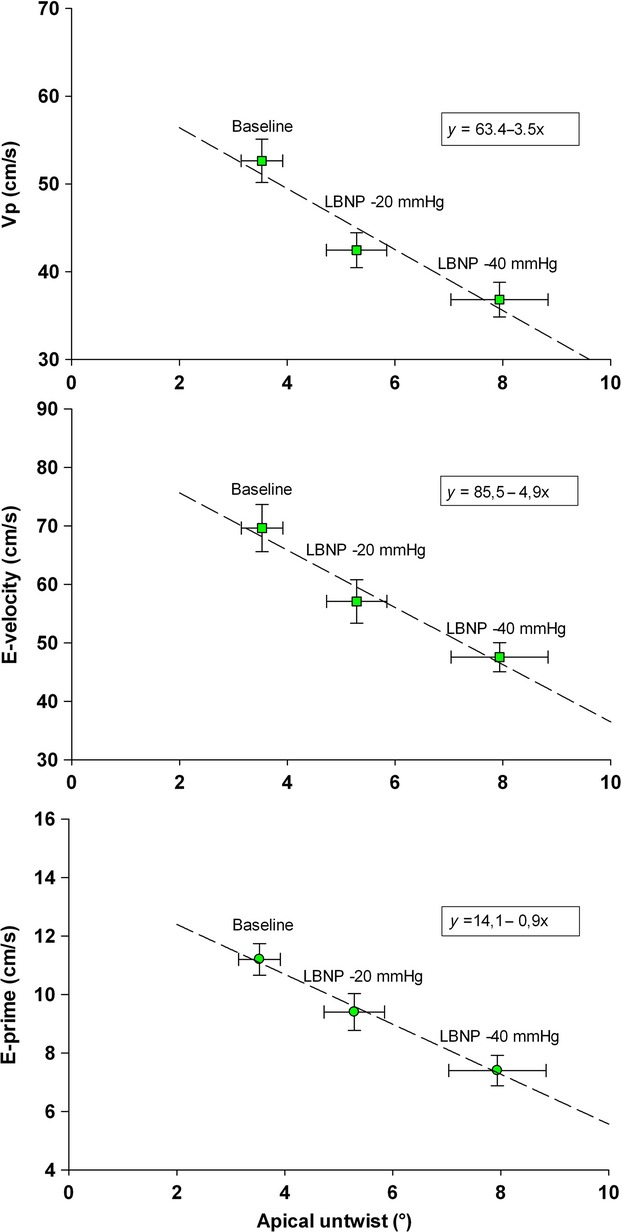
Correlation plots between average values of the 12 healthy subjects in apical untwist during IVRT and E, *V*p, and E' during early diastolic filling at rest, at lower body negative pressure (LNBP) −20 mmHg and −40 mmHg, respectively.

There were significant correlations in the 12 subjects between average values of E, *V*p, E', and apical untwist at rest, LBNP −20 and LBNP −40 mmHg (Fig.5).

### Variability

Speckle tracking echocardiography analyses of peak untwist rate during IVRT at apical level by two independent observers in all 12 subjects showed an ICC of 0.92 (0.85–0.96, *P* < 0.05).

## Discussion

The main findings in this study was that preload reduction in healthy humans was associated with increased LV regional untwist and untwist rate at subpapillary and apical level but no change at the base, resulting in an increase of net untwist and net untwist rate. To the best of our knowledge, these findings have never previously been demonstrated in humans. In addition, the increase in net untwist during preload reduction was associated with a concomitant decrease in LV flow propagation velocity, E-wave velocity of mitral inflow, and mitral annulus E' velocity. Finally, no change in E/E' ratio occurred.

### LV regional untwist

The nonuniform orientation of the myocardial fibers in the left ventricle enables a twisting motion during ejection with a clockwise rotation at the basal level and counter clockwise rotation at the apical levels (Buchalter et al. 1990; Lorenz et al. 2000; Notomi et al. 2008). At rest regional base to apex gradients with increasing counterclockwise rotation towards apex during systole have previously been demonstrated in healthy humans by MRI and with speckle tracking echocardiography (Lorenz et al. 2000; Notomi et al. 2008; Hodt et al. 2011). Furthermore, it is known that changes in preload can facilitate changes in volume, longitudinal, and radial LV geometry and strain that would invoke changes in LV twist mechanics (Arts et al. 1984; Opdahl et al. 2012). We have shown in healthy humans that preload reduction by LBNP facilitated an increase in LV base to apex gradients during systole in rotation and circumferential strain compared to rest, while longitudinal strain was reduced (Hodt et al. 2011). Regarding effects during diastole, animal studies have shown that changes in volume by abrupt reduction in preload by vena cava occlusion (first 20 heart beats) would invoke an increase in LV apical untwist or remain unchanged during IVRT (Kroeker et al. 1995; Opdahl et al. 2012). Unlike models using vena cava occlusion, the LBNP technique reflects steady-state settings 1 min after onset of constant preload reduction, allowing compensatory hemodynamic mechanisms to occur. We therefore suggest that increased apical untwist in a steady-state period during preload reduction in this study (Figs2 and 3) can be explained by increased LV inotropy modulated by similar observations that occur during adrenergic stimulation, for example, dobutamine infusion (Rademakers et al. 1992; Kroeker et al. 1995) and during LBNP (Lollgen et al. 1986; Arbab-Zadeh et al. 2004).

It is also interesting to notice that there was a markedly more increase in regional untwist rate (UTR) during IVRT than rotation rate (RR) during ejection at the subpapillary and apical levels (*P* < 0.05) (Table3). An explanation for this noticeable difference might be that untwist during IVRT provides different effects in passive and active recoil of the elastic myocardial fibers (Robinson et al. 1986; Helmes et al. 2003) between base and apex. The active recoil during IVRT seems to vary by changes in Ca^2+^ reuptake into the sarcoplasmic reticulum in the myocytes that can be regulated according to the degree of changes in inotropy and LV filling (Frank et al. 2003). At last, the anatomical orientation of the increasing oblique muscle fibers towards the apex constitute a basis for such an effective heart muscle performance (Streeter et al. 1969; Notomi et al. 2008).

Mitral to apical intraventricular pressure gradient (IVPG) has been shown to be present to move the blood from base to apex in early diastole (Courtois et al. 1988) and the corresponding blood flow can be measured as flow propagation velocity by color M-mode Doppler (Stugaard et al. 1993; Firstenberg et al. 2001a). In animals, an association between a reduction in both IVPG and mitral to apical flow propagation velocity has been shown during acute ischemic LV failure and a corresponding association between an increase in both IVPG and mitral to apical flow propagation velocity through surgical revascularization that indicate a corresponding change in elastic recoil and LV function (Steine et al. 1999; Firstenberg et al. 2001a). In this study, we have demonstrated a significant reduction of flow propagation velocity by a simultaneous increase of apical untwist and base to apex untwist gradient in a stabile hemodynamic phase at −20 and −40 mmHg by LBNP in healthy humans. At first glance, this seems to be contradictory. However, we suggest that increased untwist reflects a diastolic compensatory mechanism of the LV to maintain a sufficient IVPG during preload reduction to move the necessary amount of blood towards the apical region in order to keep up stroke volume and thus blood pressure.

### LV global net untwist

Physiological effects of changes by dobutamine on net untwist and net untwist rate have been thoroughly demonstrated in several invasive animals studies (Rademakers et al. 1992; Wang et al. 2007; Opdahl et al. 2012). These data have shown significant associations between increased net untwist and net untwist rate, shortening of the time constant (*τ*) of LV isovolumic pressure decay and reduction in end-systolic volume indicating hemodynamic changes with increased inotropy.

How changes in load affects LV net untwist and net untwist rate has been shown in a few animal and human studies (Kroeker et al. 1995; Wang et al. 2007; Park et al. 2010; Weiner et al. 2010; Opdahl et al. 2012). Using dogs, vena cava occlusion was performed transiently, and net untwist or untwist rate was measured by either an apical device (Kroeker et al. 1995), sonomicrometry (Opdahl et al. 2012), or by STE (Wang et al. 2007) for direct measurement of apical rotation. In the studies by Gibbons Kroeker et al. and Wang et al., an increased net untwist and net untwist rate was demonstrated, while Opdahl et al. found unchanged net untwist rate during IVRT. In addition, one human study has shown increased LV untwist rate during infusion of nitroprusside (Park et al. 2010). Although nitroprusside also provide a preload reduction, they speculated that the main effect was a decrease in afterload. In the human study by Weiner et al. (2010), preload was increased by a saline bolus resulting in an increase in stroke volume and net untwist. HR and BP were reported to be unchanged indicating a reduction in TPR and thereby stabilized afterload. The hemodynamic condition in Weiner et al.'s study is therefore different from ours, where TPR is increased as a consequence of both reduced preload and stroke volume by LBNP (Table1). That both changes in pre- and afterload seem to affect global net untwist is likely and are important to take into consideration. We therefore do not consider these results as contradictory.

Although these varying findings in the animal and human studies can probably be explained by different methodological approaches, they do not allow a definite answer to the overall topic. In contrast to the open-chest model by Kroeker et al. and Opdahl et al., we reduced preload by the LBNP technique in a human closed chest model, enabling controlled reduction in preload comparable to posture changes as from supine to upright position or acute loss of blood. Our results by increased net untwist and untwist rate (Fig.4) are also consistent with the closed chest and stable hemodynamic model by Wang et al. (2007).

Torsion is LV twist normalized for distance between two short axes levels and is used by others (Arts et al. 1984; Rademakers et al. 1992; Lorenz et al. 2000). Measuring LV torsion might therefore be useful as the LV ED length may change during preload reduction. In this study, the LV length from the mitral valve plane to apex at end-diastole was measured. From rest to LBNP 40 mmHg, this length was reduced in all subjects (Table1). As net twist becomes higher in all subjects, we can see from the equation, net twist/(base-apex length), that torsion, similar to net twist, is higher during preload reduction.

### Early diastolic filling

Transmitral peak E-wave velocity, mitral to apical flow propagation velocity (*V*p), and tissue velocity E' were similarly reduced from rest to LBNP −40 mmHg. Our results on E and E' are consistent with others showing the preload dependency in the healthy heart in animal studies by vena occlusion technique (Firstenberg et al. 2001b; Nagueh et al. 2001) and in humans by LBNP (Berk et al. 1990). Moreover, the ratio of E/E' that is used as a estimate of LV filling pressure did not change from rest to −40 mmHg, as a consequence of similar reduction in E and E' by preload reduction. This should emphasize, similar to others findings, that this reflects changes in LV inflow and load alterations in healthy hearts than estimating LV filling pressure (Bhella et al. 2011).

*V*p as well as E′ has been suggested as a noninvasive index for LV relaxation (Stugaard et al. 1993; Takatsuji et al. 1996; Garcia et al. 2000). The response of *V*p by preload reduction has been tested in animals and humans with conflicting results. Two animal studies have demonstrated that *V*p was not altered during preload reduction in normal dog models using vena cava constriction without changes in HR or time constant of LV isovolumic relaxation (Stugaard et al. 1993; Garcia et al. 2000). Studies in healthy humans have shown a decrease in *V*p by reduced preload by hemodialysis and LBNP (Graham et al. 2003; Prasad et al. 2007). Based on our findings, we therefore suggest that *V*p has to be looked upon just as load dependent as E and E' in healthy individuals.

### Limitations

Among the 23 screened subjects included, the image quality was found to be acceptable for further analyses in 12 subjects at apical level and 11 at basal, papillary and subpapillary level. At first glance this may seem like a possible bias. However, since STE analyses are based on measurements of changes in particular speckles in 2D images frame by frame, it requires optimal image quality to reflect physiological events at the most optimal way, which was the reason for the high exclusion rate.

The recordings showing changes in LV untwist were obtained in a hemodynamic steady-state setting allowing reduction in LV volumes and changes in geometry to occur simultaneously in addition to compensatory hemodynamic adrenergic mechanisms. Although these changes might contribute to the observed increased LV untwist, we acknowledge not to have established the cause-and-effect relationship.

Our study included only healthy young men. It will, however, be of interest to address the clinical relevance of our findings in different age groups, both sexes, different heart diseases, and particularly in patients with diastolic heart failure with preserved ejection fraction.

This study is limited to demonstrate the individual associations between changes in events during early diastolic filling parameters by E, *V*p, E', and changes in untwist and untwist rate between rest and LBNP −40 mmHg and do not address the cause-and-effect relationship between these echo indices and the LV performances.

Ideally, all measurements should be simultaneous, which was not possible to perform. However, we took care to maintain constant hemodynamic conditions during all the data recordings.

## Conclusions

Preload reduction by LBNP in healthy humans elicited increased LV regional untwist and untwist rate at apical levels during IVRT while early diastolic filling parameters were decreased. The nonuniform myocardial responses of regional untwist between base and apex during preload reduction might be physiological mechanisms to minimize the impairment in LV early diastolic filling and stroke volume and thus maintain blood pressure.

## Conflict of Interest

None declared.
